# Comparative Evaluation of Astigmatic Changes Induced by Superior and Temporal Corneal Incisions in Sutureless Phacoemulsification Surgery: A Case Series

**DOI:** 10.7759/cureus.48084

**Published:** 2023-10-31

**Authors:** Farheen Laliwala, Saloni Patel, Vaishali Prajapati, Lisa Patel, Mayur B Wanjari, Deepika Singhal

**Affiliations:** 1 Department of Ophthalmology, Gujarat Medical Education and Research Society's Medical College and Civil Hospital, Ahmedabad, IND; 2 Department of Research and Development, Jawaharlal Nehru Medical College, Datta Meghe Institute of Higher Education and Research, Wardha, IND

**Keywords:** keratometry, temporal incision, superior incision, postoperative astigmatism, phacoemulsification

## Abstract

Background

In cataract surgery, the effect of corneal incision astigmatism has been widely recognized for many years. The incision's size, shape, and location can all impact the patient's postoperative visual outcomes. Currently, phacoemulsification is considered the most preferred surgical technique for cataract extraction. However, there is still some debate about whether temporal incisions, which are smaller and considered nearly astigmatic neutral, result in more astigmatism than other incisions. As a result, it is important to continue studying the refractive changes induced by corneal incisions made at different sites during phacoemulsification surgery.

Aim and objective

To compare the incidence, extent, and course of postoperative astigmatic changes associated with superior versus temporal clear corneal incisions for sutureless phacoemulsification cataract surgery.

Materials and method

In this prospective study, 50 patients of the civil hospital in Gujrat with cataracts who underwent sutureless, small incision (2.8 mm) phacoemulsification surgery were included. The preoperative evaluation comprised visual acuity assessment, refraction, keratometry, fundus examination, and intraocular lens (IOL) power calculation. The superior incision was made in 25 patients, and the temporal incision was made in another 25 patients. Patients were examined preoperatively on day 1, at one week (day 7), after one month (day 30), and after two months (day 60).

Result

Postoperatively, two months (on day 60) postoperatively, in group A (superior approach), the mean surgically induced astigmatism (SIA) was 0.39±0.34 SD diopters, and in group B (temporal approach), it was 0.5±0.42 SD diopters. A significant statistical difference was not seen between these two groups.

Conclusion

Surgically induced astigmatism was minimal and comparable with both superior and temporal approaches to clear corneal incisions for phacoemulsification surgery.

## Introduction

Charles D. Kelman introduced the concept of phacoemulsification in 1948, but it was accepted after 1967 [1]. The surgical techniques of phacoemulsification have evolved over the years. Corneal incision-induced astigmatism has been known in cataract surgery for over a century. At present, phacoemulsification is the surgery of choice for cataract removal. In this technique, the surgery is performed through a 3 mm or smaller incision, and the lens is aspirated after phacoemulsification. In phacoemulsification surgery, a hollow 1 mm titanium needle with a piezoelectric crystal, which vibrates at an ultrasonic speed of 40,000 per second, fragments the nucleus of the cataractous lens and then emulsifies these fragments [2]. The technique also uses a surgeon-controlled automated irrigation and aspiration system to aspirate the emulsified nucleus and cortical matter [2].

Phaco wounds are of three types: sclerocorneal tunnel incisions, limbal corneal tunnel incisions, and clear corneal tunnel incisions [2]. The clear corneal incision is the most commonly used for phacoemulsification as it has less postoperative inflammation and pain and minimal surgically induced astigmatism (SIA) [3-6]. Another big advantage of clear corneal incisions is the ability to do surgery with topical anesthesia and excellent access to the anterior chamber for proper capsulorhexis performance, access to the cataract, and intraocular lens (IOL) placement. These are bloodless, self-sealing, sutureless, and quick incisions [4]. However, one of the major disadvantages of this clear corneal incision is the possibility of increased endothelial cell loss. Large wounds may be leaky, not self-sealing, and may even require sutures [2,7-9].

For clear corneal incisions, frequently used approaches are superior and temporal. The advantage of the superior approach is that an incision placed underneath the upper lid causes less discomfort in patients [10]. The surgeon’s hand can rest on the patient's forehead during the surgery. Still, the orbital edge can impair access to the surgical field, especially in deep-set eyes [2]. In the temporal approach, which provides excellent access to the surgical field, it is important to note that Bell's phenomenon, a reflex eye movement, does not pose a problem when using topical anesthesia. Additionally, the future trabeculectomy site, which is the location of a surgical procedure, remains unaffected. However, it is worth mentioning that postsurgery, frequent adjustments are necessary for the setup of both the right and left eyes of the patients [2].

Cataract surgery is known as ‘refractive surgery’ because it aims to correct preexisting refractive errors and remove cataracts [9]. A spherical refractive error can be eliminated by meticulously performing IOL power calculations. Postoperative astigmatism can be controlled by managing pre-existing and SIA [2]. The astigmatic change introduced by surgical corneal treatment is surgically induced astigmatism [2]. SIA depends upon the incision's length, location, shape, and direction. SIA must be minimal to achieve the best uncorrected visual acuity after surgery. Superior incision induces astigmatism that opposes the rule and effectively counteracts the pre-existing astigmatism. In contrast, temporal incision induces the rule of astigmatism and so neutralizes previous arguments against the rule of astigmatism [2,11].

## Materials and methods

Study setting and design

This non-randomized, quasi-experimental study was conducted at a tertiary care teaching hospital in Ahmedabad, Gujarat. An operating surgeon was randomly assigned to undertake cataract surgery by superior versus temporal clear corneal incisions for sutureless phacoemulsification.

Study population

Fifty patients with uncomplicated unilateral or bilateral senile cataracts up to grade three nuclear sclerosis were included in the study. The patients were randomly divided into two groups, with 25 in each group.

Study participant criteria

Study's inclusion and exclusion criteria are listed in Table 1.

**Table 1:** Study's inclusion and exclusion criteria

Inclusion criteria	
1	Patients with uncomplicated unilateral or bilateral senile cataracts up to grade 3 nuclear sclerosis
Exclusion criteria	
2	Patient with high preoperative astigmatism of more than 3 diopters
3	Patient with complicated cataract
4	Patients with any eye disease - glaucoma, squint, pseudoexfoliation, retinal or macular diseases, diabetic or hypertensive retinopathy, etc.
5	Suspected or positive COVID-19 patient
6	Patient with past history of any corneal surgery or corneal disease
7	Patient with past history of trauma
8	Patient with past history of any intraoperative or postoperative complication
9	Patient with any systemic illness - diabetes mellitus, hypertension, asthma, etc., or immunocompromised patient
10	Patient who lost to follow-up

Data collection

A thorough preoperative evaluation of the anterior and posterior segments was performed, including uncorrected visual acuity, best-corrected visual acuity, slit lamp examination, grading of cataract, fundus examination, auto refractive keratometry, and IOL power calculation. Patients were randomly assigned to two groups, with 25 in each group. Phacoemulsification was performed through a 2.8-mm clear corneal incision by the superior approach in group A and the temporal approach in group B. Postoperatively, all patients were given topical antibiotics and steroid combination eye drops. A complete ophthalmological examination was performed on day 1, day 7, day 30, and, at last, day 60.

Statistical analysis

The astigmatism of both groups was compared using the SIA calculator version 2.1. Statistical analysis was performed using SPSS software (IBM Corp., Armonk, NY). Descriptive statistics were used to summarize the data, and a t-test was used to compare the mean SIA between the two groups.

## Results

Table 2 shows that the most common age group in both groups was 56-60. The patient's mean age in group A was 54±5.23 SD years and in group B was 56±4.73 years.

**Table 2:** Age distribution of patients in the study

Age group (years)	No. of patients (group A)	No. of patients (group B)
41−45 years	2	1
46−50 years	5	3
51−55 years	6	6
56−60 years	9	10
61−65 years	3	5

Table 3 shows that out of 25 patients in group A, 15 were male and 10 were female. Of the 25 patients in group B, 14 were male and 11 were female. Out of 50 patients, males were 29 (58%), and females were 21 (42%), with a male-to-female ratio of 1.3:1.

**Table 3:** Gender distribution of patients in the study

Gender	No. of patients (group A)	No. of patients (group B)
Male	15(60%)	14(56%)
Female	10(40%)	11(44%)

Table 4 shows that the mean preoperative astigmatism in group A was 0.46D±0.52 SD diopters, and in group B it was 0.45D±0.48 SD diopters. In group A, the mean postoperative astigmatism on day 1 was 0.77±0.54 SD diopters, gradually reducing to 0.49±0.36 SD diopters on day 60. In group B, the mean postoperative astigmatism on the day was 0.89±0.68 SD diopters, which gradually reduced to 0.55±0.51 SD diopters on day 60.

**Table 4:** The pre and postoperative astigmatism on days 1, 7, 30, and 60

Astigmatism (diopters)	No. of patients (% of patients)									
	Pre-op		Post-op day-1		Post-op day-7		Post-op day-30		Post-op day-60	
	Group -A	Group -B	Group- A	Group- B	Group -A	Group- B	Group- A	Group- B	Group- A	Group -B
0	12(24%)	10(20%)	5(10%)	6(12%)	5(10%)	6(12%)	6(12%)	6(12%)	6(12%)	7(14%)
0.25	1(2%)	1(2%)	2(4%)	0(0%)	2(4%)	0(0%)	2(4%)	5(10%)	3(9%)	5(10%)
0.5	2(4%)	6(12%)	2(4%)	4(8%)	6(12%)	5(10%)	7(14%)	4(8%)	7(14%)	4(8%)
0.75	4(8%)	4(8%)	4(8%)	3(6%)	4(8%)	2(4%)	5(10%)	1(2%)	4(8%)	2(4%)
1.0	3(6%)	2(4%)	6(12)%	3(6%)	6(12%)	4(8%)	5(10%)	4(8%)	5(10%)	3(6%)
1.25	1(2%)	1(2%)	1(2%)	0(0%)	1(2%)	0(0%)	0(0%)	1(2%)	0(0%)	1(2%)
1.5	2(4%)	0(0%)	5(10%)	4(8%)	1(2%)	5(10%)	0(0%)	4(8%)	0(0%)	3(6%)
1.75	0(1%)	1(2%)	0(0%)	4(8%)	0(0%)	1(2%)	0(0%)	0(0%)	0(0%)	0(0%)
2.0	0(0%)	0(0%)	0(0%)	1(2%)	0(0%)	0(0%)	0(0%)	0(0%)	0(0%)	0(0%)
Mean astigmatism	0.46	0.45	0.77	0.89	0.61	0.73	0.51	0.61	0.49	0.55
SD	±0.52	±0.48	±0.54	±0.68	±0.42	±0.57	±0.36	±0.54	0.36	±0.51

Table 5 shows the rule: astigmatism preoperatively was 22% in group A and 26% in group B, while postoperatively, it was present at 20% and in group B at 28%, respectively. Against the rule, astigmatism in both preoperative groups was 4%, while postoperatively, in group A, it was 18%, and in group B, it was 8%. On postoperative day 60, the mean SIA in group A was 0.39±0.34 SD diopters and 0.5±0.42 SD diopters in group B. The difference between the two groups was insignificant (p-value=0.27).

**Table 5:** Measurements were taken for both with-the-rule and against-the-rule astigmatism, both pre- and postoperatively, on days 1, 7, 30, and 60

	Preoperative		Postoperative	
	Group A	Group B	Group A	Group B
Nil	12(24%)	10(20%)	6(12%)	7(14%)
With the rule	11(22%)	13(26%)	10(20%)	14(28%)
Against the rule	2(4%)	2(4%)	9(18%)	4(8%)

## Discussion

Both groups had minimal surgically induced astigmatism after phacoemulsification and foldable IOL implantation. With the presentation of phacoemulsification, SIA has decreased through a smaller, clear corneal incision. In the present study, the most common age group in both groups was 56-60 years, and out of 50 patients, 29 (58%) were male and 21 (42%) were female. In a study by Yoon et al. [4], the mean age of the patients was 66.2±7.6 years, and there were 17 men and 13 women out of 30 patients [4].

In the present study, the mean preoperative astigmatism in group A was 0.46±0.52 SD diopters, and in group B it was 0.45±0.48 SD diopters. In a study by Nikose et al. [2], in both groups, the largest number of patients had astigmatism in the range of 1-2 preoperatively [2]. In a study by Harakuni et al. [5], in 32% of patients, preoperative astigmatism was 0.5 D; in 51% of patients, it was in the range of 0.5-1 D; and in 17% of patients, it was in the range of 1-1.5 D [5].

In the present study, the mean postoperative astigmatism on day 60 was 0.49±0.36 SD diopters in group A (superior approach) and 0.55±0.51 SD diopters in group B (superior approach). Nikose et al. [2] suggest that the superior approach group had an average postoperative astigmatism of around 1.5 diopters, whereas the temporal approach had around 1.0 diopters [2].

In the present study, on postoperative day 60, the mean SIA in group A (superior approach) was 0.39±0.34 SD diopters and in group B (temporal approach) was 0.5±0.42 SD diopters. In a study by Nikose et al. [2], the mean SIA in the temporal group was 0.768 diopters, and the superior group was 1.293 diopters after 90 days. In a study by Harakuni et al., the SIA was 0.70±0.35 D in a temporal incision group and 0.84±0.49bD in a superior incision group [5]. In a study by Marek et al. [6], surgery with a temporal incision group had a mean SIA of 0.63±0.28 D, and surgery with a superior incision group had a mean SIA of 1.00±0.54 D. While in a study by Giasanti et al. [7] in postoperative (three-month) evaluation, the differences were not significant [7].

Limitation of the study

As the study's sample size is limited, the results cannot be generalized. A multicentric study with more study participants will be needed to get this technique's actual picture and limitations.

## Conclusions

The present study concluded that the surgically induced astigmatic changes or changes in preoperative astigmatism are minimal and comparable with both approaches - superior and temporal clear corneal incisions for phacoemulsification cataract surgery. With surgical experience and proper surgical techniques, both "temporal and superior" clear corneal incisions can lead to optimum uncorrected visual acuity after phacoemulsification surgery. Flattening of the cornea occurs at a right angle to the incision during the superior incision. Cataract surgery, so, against the rule, astigmatism is more common in the superior approach. While the approach is away from the visual axis in the temporal incision, cataract surgery, with the rule, astigmatism’ is more common in the temporal approach.
